# The risks of using molecular biodiversity data for incidental detection of species of concern

**DOI:** 10.1111/ddi.13108

**Published:** 2020-08-13

**Authors:** John A. Darling, Xavier Pochon, Cathryn L. Abbott, Graeme J. Inglis, Anastasija Zaiko

**Affiliations:** 1 Center for Environmental Measurement & Modeling, United States Environmental Protection Agency, Research Triangle Park, NC, USA; 2 Coastal and Freshwater Group, Cawthron Institute, Nelson, New Zealand; 3 Institute of Marine Science, University of Auckland, Warkworth, New Zealand; 4 Department of Fisheries and Oceans, Pacific Biological Station, Nanaimo, British Columbia, Canada; 5 National Institute of Water & Atmospheric Research Ltd., Christchurch, New Zealand; 6 Marine Research Institute, Klaipeda University, Klaipeda, Lithuania

**Keywords:** biodiversity monitoring, biosecurity, data quality, high-throughput sequencing, incidental detection, species of concern

## Abstract

Incidental detection of species of concern (e.g., invasive species, pathogens, threatened and endangered species) during biodiversity assessments based on high-throughput DNA sequencing holds significant risks in the absence of rigorous, fit-for-purpose data quality and reporting standards. Molecular biodiversity data are predominantly collected for ecological studies and thus are generated to common quality assurance standards. However, the detection of certain species of concern in these data would likely elicit interest from end users working in biosecurity or other surveillance contexts (e.g., pathogen detection in health-related fields), for which more stringent quality control standards are essential to ensure that data are suitable for informing decision-making and can withstand legal or political challenges. We suggest here that data quality and reporting criteria are urgently needed to enable clear identification of those studies that may be appropriately applied to surveillance contexts. In the interim, more pointed disclaimers on uncertainties associated with the detection and identification of species of concern may be warranted in published studies. This is not only to ensure the utility of molecular biodiversity data for consumers, but also to protect data generators from uncritical and potentially ill-advised application of their science in decision-making.

High-throughput sequencing (HTS) is revolutionizing our ability to characterize biodiversity across ecosystems. Recent technological advances have enabled researchers to extract information about entire biological communities by generating DNA sequence data derived from bulk environmental samples, comparing those sequences to rapidly expanding reference databases, and ultimately inferring the presence of particular taxonomic groups, often with species-level resolution (Taberlet, Bonin, Zinger, & Coissac, 2018). These methods offer unprecedented opportunities for new species discovery, ecological trends monitoring and environmental impact assessment (Hunter, Hoban, Bruford, Segelbacher, & Bernatchez, 2018; Pawlowski et al., 2018). They also raise the possibility of dramatically enhancing incidental detection of species of concern (SOC), including invasive and pathogenic species and threatened, endangered and other vulnerable species (Jarrad et al., 2011; Prins & Kok, 2016). In contrast to active surveillance, in which a target species is deliberately sought using highly specific and sensitive tools already proven to be fit-for-purpose, incidental detection is *the unanticipated detection of SOC in the context of a broader survey*. This approach may allow early detection of new unanticipated incursions or determination of the presence of extremely rare and ephemeral species. It could also enable efficient leveraging of resources, with HTS applications in a variety of biodiversity monitoring contexts being adopted secondarily as opportunities for SOC surveillance.

Unfortunately, there are significant and possibly underappreciated risks to incidental HTS-based detections. Specifically, data quality and assessment criteria required in surveillance contexts—where the detection of SOC could have costly management, trade, or health implications and may even be exposed to legal challenge—are exceptionally rigorous and rarely met in broader biomonitoring settings (Darling & Mahon, 2011). This scrupulousness is due in large part to concerns regarding the possibility of false-positive detections (the identification of a taxon that is not actually present in the surveyed habitat), the occurrence of which can result in misapplication of scarce resources and erosion of public confidence in surveillance programmes. Unfortunately, false-positive errors may arise at multiple points in the HTS workflow from sample collection through bioinformatic analysis and can be extremely challenging to detect in the absence of exacting quality controls or detailed post hoc analysis of taxonomic assignments (Cristescu & Hebert, 2018). The approaches to quality assurance commonly applied in standard HTS biodiversity surveys generally do not meet these demanding standards, and associated data therefore may not be fit-for-purpose in surveillance contexts.

These problems are exacerbated by the exceedingly broad taxonomic coverage available through HTS methods and their evergrowing application across biomes. Although incidental detections during general biomonitoring have always been possible with traditional methods, HTS technology has dramatically altered the monitoring landscape by offering access to diversity across the taxonomic spectrum. HTS biodiversity assessments may therefore capture components of biological communities previously not accessed by non-targeted efforts—specifically, parasitic and pathogenic taxa, many of which pose direct risks for agricultural resources or human populations (Sekse et al., 2017). Indeed, recent developments in global public health related to pandemic response dramatically highlight the potential value of early detection of such SOC. Yet the reliability, robustness and reproducibility of HTS survey data have typically not been formally characterized, leaving error rates largely unknown (Deiner et al., 2017) and their utility for SOC surveillance unproven.

The fundamental problem in the current research climate is that there are no widely established quality criteria or reporting standards to determine which species records from HTS-based biomonitoring studies are fit-for-purpose in surveillance contexts. Potential end users of these data therefore have little basis for determining if the report of a particular SOC has been verified with sufficient confidence to warrant consideration in decision-making. Nevertheless, efforts to catalogue biodiversity using HTS have advanced into almost every conceivable ecological milieu and the number of species lists published based on HTS analyses promises to escalate dramatically in coming years. These lists will exist side by side with policy triggers, many of which are legally enforceable and mandate action based on the presence of SOC, and it is not clear how they might be dealt with by consumers who have historically had to make policy decisions in data-poor environments. Indeed, public reporting is often considered a valuable avenue for detecting SOC; in biosecurity contexts, for instance, reporting new incursions of unwanted SOC may be actively encouraged or even imparted as a duty in some jurisdictions. In New Zealand, sections 44 and 46 of the *Biosecurity Act 1993* oblige “every person” to report the presence of apparently new or notifiable organisms to the Ministry for Primary Industries (MPI). But despite its value to surveillance, public reporting is burdened by high rates of false alarms that consume limited resources (Froud, Oliver, Bingham, Flynn, & Rowswell, 2008), a problem that may be exacerbated by incidental HTS-based detections. These challenges will likely be further aggravated by efforts to harness “big data” approaches to biodiversity science, which may enlist automated or artificial intelligence-empowered algorithms for populating biodiversity databases from published records (Kays, McShea, & Wikelski, 2019).

The lack of clear distinction between HTS data produced for general biodiversity studies and those produced specifically for SOC surveillance is clearly a challenge for biosecurity end users, particularly given that many may lack the technical knowledge to appraise HTS methods and interpret the quality of results. Perhaps less obvious is that generators of HTS-based biodiversity data should be aware of the possibility of unwittingly publishing a species name that could be used injudiciously in a decision-making context, with uncertain consequences for both third-party stakeholders (e.g., industries that might be affected by discovery of SOC) and the authors themselves. It is therefore critically important that researchers be sensitive to the possibility of reporting SOC in their HTS datasets, and be prepared for the heightened scrutiny imposed when their data are interpreted in the context of surveillance applications.

The context for decision-making related to biosecurity response is inherently challenging both politically and emotionally; decisions must be made rapidly with limited data and may have highly unfavourable implications for various stakeholders. Appropriate vigilance around the quality of the data used to make decisions is vital. Unfortunately, there have been instances where identification of SOC in published HTS datasets has elevated management concerns and been later proved erroneous by additional *post hoc* quality control steps on already published data (Afshinnekoo et al., 2015a, 2015b). Analysis of a small sample from the literature in our own research field (marine biodiversity) indicates that publishers of HTS biodiversity data may be insufficiently cognizant of these risks. Only rarely do publications appear to explicitly recommend caution in accepting HTS-based taxonomic assignments, even in cases listing taxonomic groups that potentially include SOC (Figure 1). Although this analysis represents only ~10% of relevant papers in the field, it is illustrative of a norm in published work to present HTS-derived biodiversity inventories without clearly cautioning prospective end users regarding the potential for false-positive error.

A case in point from New Zealand also highlights this concern. In 2018, a PhD project aimed at methodological advances for HTS monitoring of marine biodiversity recovered a single sequence from a pathogenic species notifiable to the World Organization of Animal Health and subject to management by MPI (Lane, Webb, & Duncan, 2016). Fortunately, an advisor on the project recognized the potential issue and urged additional rigorous testing to confirm the taxonomic assignment prior to publication; those tests indicated that the initial assignment, conducted to commonplace data quality standards, was inaccurate, and the species list was updated accordingly prior to publication (Ammon et al., 2018). If not for this intervention, the final publication would have identified the SOC as a new incursion to New Zealand’s North Island. At that time, the pathogen was under management in part of the South Island with a Controlled Area in place that presumed its absence from other areas. It is unknown whether this mistaken identification would have triggered a management response, but it is plausible that additional concern and expenditure of resources may have resulted, with potentially uncomfortable implications for the authors of the study as well as impacted industries.

These examples suggest that researchers conducting broad biodiversity surveys based on HTS data often overlook the possibility that their results may be of considerable interest to end users concerned with SOC. There are multiple potential reasons for this apparent insensitivity, including a lack of accessible tools for recognizing SOC when they show up in diversity inventories and widely acknowledged pressures to rapidly publish impactful studies. Regardless, there is a clear need to develop and adopt standardized quality control and reporting criteria for HTS studies to enable appropriate evaluation of species detections by potential end users. With the implementation of, and adherence to, these criteria, assignations of genuine SOC could be flagged, allowing end users access to data known to be fit-for-purpose to aid management decision-making and enable appropriate responses. Though this arrangement would not eliminate nefarious attempts to mis-use scientific data or policy disputes rooted in genuine scientific uncertainty, it would limit errors made by those seeking to utilize the outputs of these studies in good faith. Figure 2 briefly sketches a vision of this path, leading from the “current state” (top) to a “desired future” (bottom) in which fit-for-purpose data can be readily recognized by data consumers in surveillance contexts and applied appropriately to decision-making.

There is already broad international acknowledgement of the need to move towards standard best practices for analysing and reporting HTS-derived biodiversity data (Ten Hoopen et al., 2017), with the aim of achieving greater reproducibility and end-user acceptance of these tools. Further ensuring that data are fit-for-purpose in surveillance settings will necessitate additional criteria reflecting the essentially forensic nature of this science. Such criteria must include standards associated with sample handling and processing, chain of custody, and limitation or estimation of potential error in both molecular and bioinformatics workflows (“transitional changes” in Figure 2); all are currently beyond the requirements typically set for general ecological studies. Studies meeting these criteria must be expected to be sensitive to existing mechanisms that tie the presence of SOC to management action. Associated with this would be additional requirements aimed at confirming taxonomic assignments from HTS data indicating the detection of SOC and strict reporting of the quality assurance standards implemented.

The development of HTS reporting criteria could lean on standards used for other diagnostic applications (e.g., international standards for detection of human and animal pathogens; Belak & Granberg, 2018) but must conform to peculiarities of HTS biodiversity data to benefit both data producers and users. Consensus on standards that allow clear identification of data fit for SOC surveillance applications will necessitate close collaboration between scientists and various stakeholders, including both consumers of surveillance data and those entities (typically governments and intergovernmental organizations) best positioned to formalize, disseminate, and even enforce data quality and reporting standards.

As the research community moves towards such standards, interim solutions are needed (“initial changes” in Figure 2). More pointed disclaimers on the uncertainties associated with taxonomic assignments made in HTS studies and the fitness of published data for end users are warranted and could become standard for peer-reviewed publications. Further, the development and wide public accessibility of regional species watch lists would greatly facilitate awareness of impending issues. Straightforward informatics tools, such as apps that screen pre-publication HTS datasets for SOC in georeferenced samples, could be readily implemented and allow researchers to flag results warranting important secondary quality assurance steps (Lammers, Peelen, Vos, & Gravendeel, 2014). For example, the United States Geological Survey is currently beta testing an online tool called Screen and Evaluate Invasive and Non-native Data (SEINeD), which will screen any georeferenced species list against the actively maintained Non-indigenous Aquatic Species database for the United States, flagging species known to be invasive or introduced to the area where data were collected (Wesley Daniels, personal communication). To secure actionable taxonomic assignments of unwanted species from HTS data, implementation of tools like this must be underpinned by quality-assured (and ideally morphologically vouchered) reference sequence databases of SOC and their close relatives, curated by appropriate expertise and with enduring support from governmental agencies.

It may be tempting for researchers to adopt a “buyer beware” approach to this issue, placing all onus on end users to consume HTS-derived biodiversity data only with caution and to resist the assumption that SOC identified in these data represent actionable observations. But so long as the research community continues to cite incidental detections of SOC as a fundamental benefit of HTS-based monitoring, it would be disingenuous to simultaneously deny responsibility for such detections when they arise. Given decision-makers’ appetite for information on SOC distributions and the potential risks associated with consumption of HTS data that are not fit-for-purpose, it should be the concern of all researchers—including even those not explicitly engaged in SOC-related studies—to rapidly move towards criteria that enable clear separation of methods appropriate for surveillance from those acceptable in general biodiversity monitoring contexts.

## Figures and Tables

**FIGURE 1:**
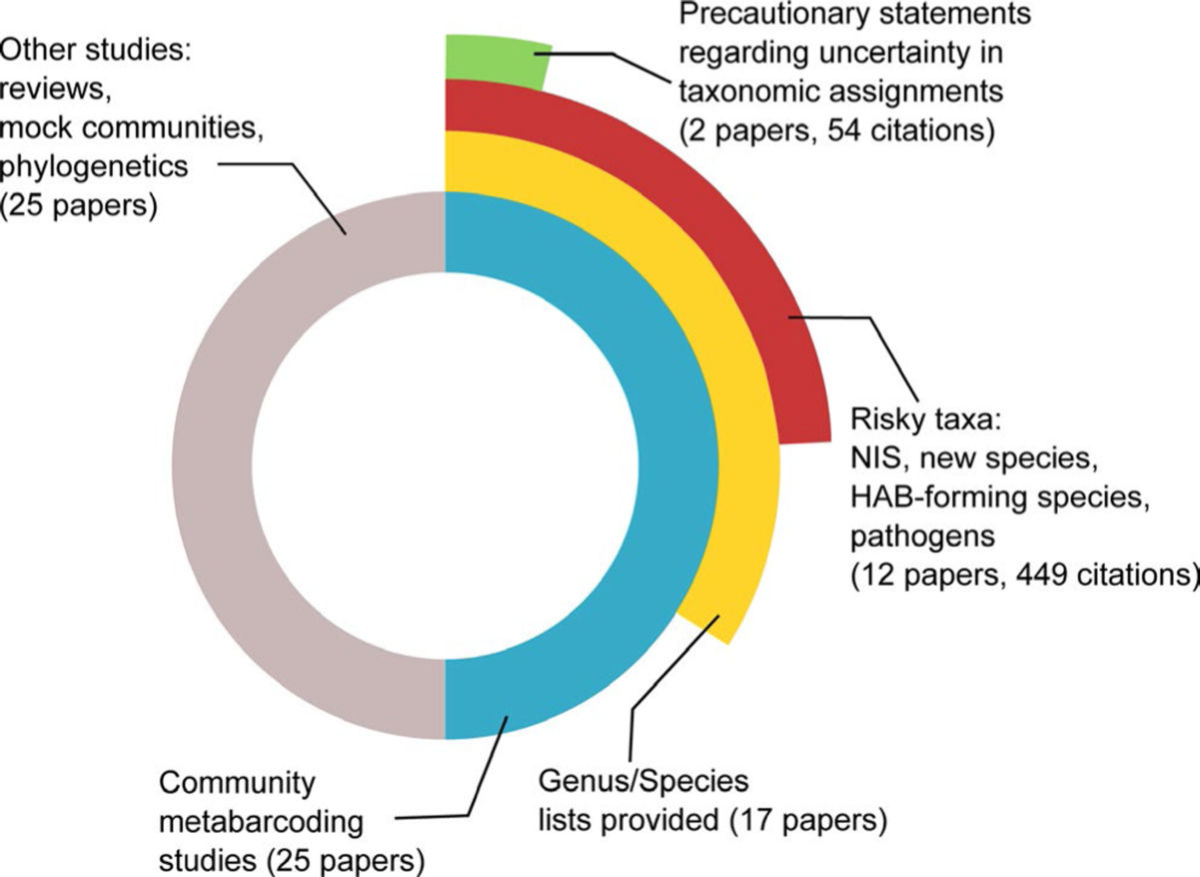
Few published papers offer precautionary statements regarding molecular identification of potential SOC. Published papers in the field of marine biodiversity studies were extracted from the ISI Web of Science Database on 22 July 2019 using the following search parameters: TS = (metabarcoding OR HTS OR NGS OR “high-throughput sequencing” OR “next generation sequencing”) AND TS = (marine OR coastal) NOT TS = (bacteria* OR microb* OR prokaryot*) AND DOCUMENT TYPES: (Article) Timespan: Last 5 years. This search returned 444 papers. Fifty of these were randomly selected for detailed investigation. First, they were screened for publications that included lists of taxa assigned at the genus and species level. These lists were further screened for the presence of potential undesirable SOC—non-indigenous species (NIS), species capable of forming harmful algal blooms (HABs) or pathogens—or genera that contain such species using a custom R script that cross-referenced species lists against online databases of known SOC. All papers providing lists of taxa at the genus/species level were also assessed for explicit statements in the main text recognizing the possibility of mis-assignment of taxonomic identity in the HTS informatics pipeline or other sources of false-positive error

**FIGURE 2:**
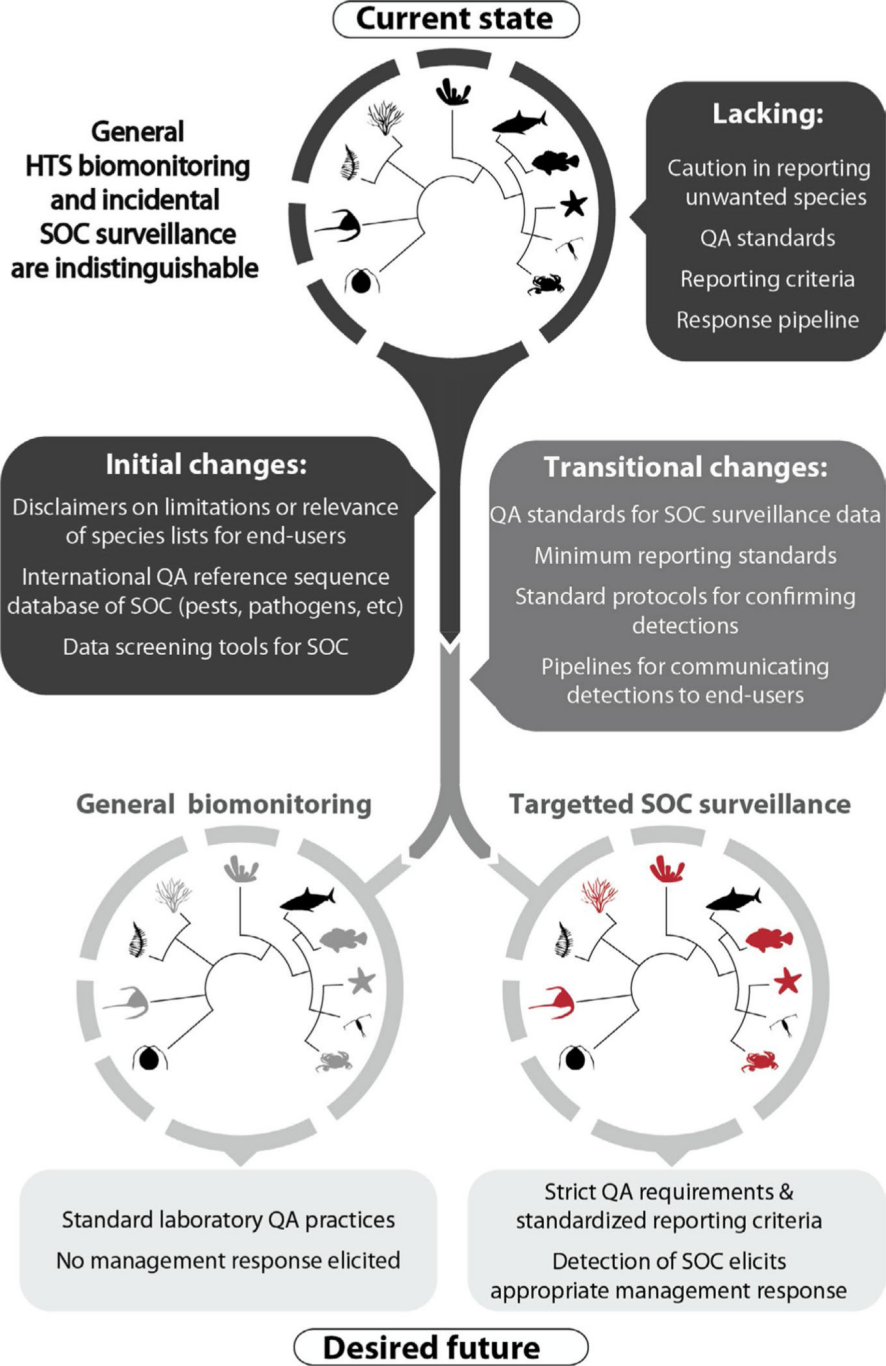
Path towards separating HTS-based surveillance from general HTS-based biomonitoring. Future data quality and reporting standards must provide a clear means of distinguishing data fit-for-purpose in SOC surveillance contexts from those generated in the course of general ecological studies. The current absence of such criteria potentially presents serious risks associated with out-of-context use of HTS data
